# Virtual reality assessment of cognitive performance in adults with ADHD and problematic internet use: a pilot study

**DOI:** 10.3389/fpsyg.2026.1788715

**Published:** 2026-06-29

**Authors:** Carmela Mento, Clara Lombardo, Federica Arena, Vincenzo Messina, Giulia Fangano, Federica Zuccalà, Fabrizio Turiaco, Angela Alibrandi, Clemente Cedro

**Affiliations:** 1 Department of Biomedical and Dental Sciences and Morphofunctional Imaging, University of Messina, Messina, Italy; 2 Psychiatric Unit, Polyclinic Hospital, Messina, Italy; 3 Department “Scienze della Salute”, University of Catanzaro, Catanzaro, Italy; 4 Unit of Statistical and Mathematical Sciences, Department of Economics, University of Messina, Messina, Italy

**Keywords:** ADHD, cognitive monitoring, executive functioning, pilot study, problematic internet use, virtual reality assessment

## Abstract

**Introduction:**

Attention-deficit/hyperactivity disorder (ADHD) is associated with variability in attentional and executive functioning, and individuals with ADHD frequently present problematic patterns of internet use. Virtual-reality (VR)–based assessments have been proposed to improve ecological evaluation of cognitive performance. This pilot study explored cognitive performance variations in adults with ADHD, examining associations with problematic internet-use during routine clinical treatment.

**Methods:**

A pilot study was conducted in 17 adults with ADHD and clinically relevant problematic internet use. Participants were evaluated at baseline (T0) and after 4 weeks (T1) using the Barkley Adult ADHD Rating Scale-IV (BAARS-IV), the Internet Addiction Test (IAT), and an immersive VR continuous performance test (Nesplora Aquarium) assessing attentional and executive-function indices.

**Results:**

Comparison between T0 and T1 revealed a significant improvement in working memory (Z = −2.638, *p* = 0.008) and a reduction in IAT scores (Z = −2.392, *p* = 0.017). Conversely, attention scores showed a decline (Z = −2.899, *p* = 0.004). At baseline, a positive correlation was found between IAT scores and working memory, which disappeared after treatment.

**Discussion:**

These results provide preliminary evidence supporting the feasibility of VR-based cognitive monitoring in adults with ADHD and require confirmation in larger controlled studies.

## Introduction

Attention deficit/hyperactivity disorder (ADHD) in adulthood is a neurodevelopmental disorder associated with persistent deficits in attention and executive functions (APA, 2013). It has a significant impact on quality of life and daily functioning (Faraone et al., 2015; Faraone et al., 2021). Assessing cognitive difficulties in ecologically valid conditions remains a significant clinical challenge, even though several neuropsychological instruments are commonly used to assess these functions. Traditional continuous performance tests (CPTs), widely used to assess sustained attention and inhibitory control, are typically administered in highly controlled environments and are unable to simulate the multisensory complexity of real-life contexts in which individuals with ADHD often experience greater functional difficulties (Huang-Pollock et al., 2012; Willcutt et al., 2005). In particular, attention disorders in ADHD tend to manifest more clearly when there are distractors and competing cognitive demands simultaneously. This highlights the need for assessment tools that can simulate more complex cognitive environments than traditional experimental settings. Virtual reality (VR) technologies in neuropsychological assessment could improve the ecological validity of cognitive tasks. Immersive environments allow for the controlled presentation of complex visual and auditory stimuli and enable the continuous recording of behavioral parameters such as reaction times, errors, and distraction (Neguț et al., 2016). Tools such as Nesplora Aquarium, which are based on virtual reality and CPT paradigms, allow for the simultaneous assessment of multiple attentional executive indices, such as vigilance, inhibitory control, and working memory, in simulated scenarios characterised by ecologically meaningful distractors (Climent et al., 2021). The use of virtual reality-based continuous performance tasks in the assessment of ADHD has been increasingly studied. Studies using immersive classroom or ecological simulation paradigms have found that virtual environments may be more sensitive to attentional variability and distraction than traditional CPTs (Fang et al., 2019). Evidence indicates that virtual reality-based assessments can differentiate ADHD attention control and inhibition difficulties while remaining effective and tolerable in individuals undergoing treatment. In particular, studies using the Nesplora Aquarium system have shown that VR-derived performance indices significantly predict ADHD symptom severity and exhibit adequate psychometric properties (Areces et al., 2019; Di Nardo, 2025). However, evidence on the use of VR tools for longitudinal cognitive monitoring is still limited, and most available studies have focused on cross-sectional diagnostic applications.

Adults with ADHD are also more susceptible to patterns of digital technology use, according to multiple studies. Problematic internet use can be thought of as part of behavior related to impulsivity, emotional dysregulation, and a tendency to seek immediate gratification, all of which are common characteristics in people with ADHD (Mento et al., 2025; De Stefano et al., 2022). Rather than representing a specific diagnostic category, this behavior can be considered a behavioral characteristic (Ioannidis et al., 2022; Wang et al., 2017; Augner et al., 2023). It has been hypothesised that these behaviors are maladaptive coping strategies or compensatory mechanisms linked to executive dysfunction and impaired inhibitory control (Brand et al., 2019; Li et al., 2016). However, the global literature continues to debate the nosological definition and classification of these behaviors. The Interaction of Person–Affect–Cognition–Execution (I-PACE) model is one of the integrative theoretical models suggesting that interactions between individual vulnerabilities, affective and cognitive processes, and lack of executive functions may help maintain ineffective patterns of technology use (Brand et al., 2016; Brand et al., 2019). However, it is difficult to test these models empirically, particularly in clinical studies using small samples. This justifies the use of exploratory methods focused on observing cognitive correlations rather than directly testing theoretical models. The use of virtual reality in neurodevelopmental disorders is becoming increasingly popular. However, there is little evidence on the use of VR tools to monitor cognitive changes in adults with ADHD during clinical treatment. In particular, few studies have examined whether neurocognitive indices created in immersive contexts are sensitive enough to identify short-term changes that occur during routine clinical care. This gap limits the understanding of VR not only as a diagnostic instrument but also as a potential tool for clinical monitoring. Based on these considerations, the current pilot study adopted a framework where pharmacological treatment (Methylphenidate, MPH) is viewed as a modulator of neuroplasticity (Quintero et al., 2022). By targeting the prefrontal-striatal circuits essential for attentional control, MPH is viewed as providing the ‘correct tools’ for adequate cognitive functioning (Coghill et al., 2014; Kowalczyk et al., 2023; Liu et al., 2025). This pharmacological stabilization is intended to optimize experiential learning, allowing participants to navigate daily challenges with the executive resources necessary to process and reinforce adaptive behavioral strategies (Fuermaier et al., 2017). Using virtual realitybased assessment, we examined changes in cognitive indices to explore how the stabilization of attentional circuits may optimize experiential learning in a sample of adults undergoing routine clinical care. Specifically, the study sought to: (1) assess changes in cognitive performance using the Nesplora Aquarium immersive assessment system between short-term follow-up and baseline; (2) thoroughly analyse the association between neurocognitive indices and the severity of problematic Internet use, which is a common behavioral characteristic in individuals with ADHD; and (3) evaluate the feasibility and sensitivity of VR-based tools for monitoring of cognitive trajectories in real-world clinical settings. The analyses were conducted for descriptive and exploratory purposes because the study was preliminary and the sample size was small. No causal relationships were assumed between drug treatment, cognitive functioning, or technology use behaviors.

## Materials and methods

### Study design and population

This pilot study was conducted at the Adult Neurodevelopmental Disorders Outpatient Clinic, University Hospital of Messina, between January and November 2024. Seventeen adults (mean age = 27.05 years, SD = 8.22; 10 males, 58.82%) with a clinical diagnosis of ADHD and clinically relevant levels of problematic internet use were enrolled and completed the full assessment protocol. ADHD diagnosis was established through specialist psychiatric assessment according to DSM-5 criteria. Clinical characterization included the evaluation of ADHD symptomatology, functional impairment, psychiatric history, and current clinical presentation as part of routine diagnostic assessment. The Barkley Adult ADHD Rating Scale-IV (BAARS-IV) was administered to quantify current symptom severity and associated impairment and to support the clinical characterization of the sample. Problematic internet use was assessed using the Internet Addiction Test (IAT), which was employed as a dimensional measure of problematic internet-use severity. Eligibility required IAT scores above the predefined threshold for clinically relevant problematic internet use (see Measures). Given the exploratory nature of the study and the limited sample size, clinical and demographic variables were used primarily for sample characterization and were not included as independent predictors in the statistical analyses. Exclusion criteria included major neurological disorders (e.g., epilepsy, traumatic brain injury) and conditions likely to invalidate cognitive testing. The study did not include a control group and adopted a within-subject pre–post design. After baseline assessment (T0), participants initiated methylphenidate treatment as part of routine clinical care. Followup assessment (T1) was scheduled 4 weeks after T0 to capture short-term variations in performance on VR-derived attentional/executive indices and self-reported internetuse severity during the early treatment period. The study was not intended to isolate medication-specific effects from non-specific factors (e.g., practice effects), and findings were interpreted as changes observed over time during routine treatment. Following baseline assessment (T0), all participants initiated treatment with methylphenidate as part of routine clinical care at the outpatient service. Methylphenidate was prescribed in a modified-release formulation and administered once daily in the morning after breakfast. Treatment was initiated at the minimum daily dose of 10 mg/day. Subsequent titration was performed by the treating psychiatrist according to standard clinical practice, considering clinical response, individual tolerability, adverse events, and patient-specific characteristics. The therapeutic aim was to identify the lowest dose associated with adequate clinical benefit. Medication management was not standardized or manipulated by the study protocol, and pharmacological parameters were not analyzed as independent experimental variables. None of the participants was receiving concomitant pharmacological treatments expected to interfere with methylphenidate action or with performance on cognitive testing. The 4-week interval between T0 and T1 was selected to observe short-term changes in VR-derived cognitive performance and self-reported problematic internetuse severity during the early phase of routine treatment adjustment. Accordingly, the observed changes were interpreted as variations occurring during clinical management and not as causal evidence of a specific medication effect.

### Measures

Assessment was conducted using the following instruments:

- The Barkley Adult ADHD Rating Scale IV (BAARS-IV) (Barkley, 2011) was used as a dimensional self-report measure to quantify ADHD symptom severity and functional impairment in adulthood and to support clinical characterization of the sample; it was not used as a stand-alone diagnostic instrument. The selfreport questionnaire assesses ADHD symptoms experienced during the previous six months using a 4-point Likert scale (1 = rarely or never; 4 = very often). The instrument evaluates four symptom domains: inattention, hyperactivity, impulsivity, and sluggish cognitive tempo, and includes an assessment of functional impairment across five contexts (home, social relationships, school, community, and work). Raw scores are converted into percentile scores based on normative data, with scores at or above the 95th percentile considered clinically significant. The BAARS-IV demonstrates strong psychometric properties in adult populations, with internal consistency coefficients ranging from *α* = 0.89 to α = 0.97 across symptom domains and high test–retest reliability. Evidence of construct validity supports the multidimensional structure of ADHD symptom clusters, while convergent validity has been demonstrated through strong associations with clinician-rated ADHD assessments and diagnostic interviews (Barkley, 2011).
- The Nesplora Aquarium (Climent et al., 2021) is an immersive virtual reality continuous performance test designed to assess attentional processes and executive functioning in individuals aged 16 to 90 years. Participants perform tasks within a simulated aquarium environment while exposed to ecological distractors, allowing objective measurement of attentional and executive indices including sustained attention, vigilance, reaction time, inhibitory control, switching ability, perseveration, and working memory through dual-execution tasks involving visual and auditory stimuli. Results are standardized using Tscores and percentiles derived from normative data. Psychometric validation was conducted on a representative normative sample of 1,469 individuals. Reliability indices were consistently high, with all task scores exceeding 0.90 (range = 0.926–0.929), and particularly strong coefficients for dual-execution conditions (Task 1 = 0.975; Task 2 with interference = 0.968). Evidence of validity includes excellent content and domain representation and convergent validity with established attentional measures such as the Caras Test (r ≈ 0.835), the D − 2 Test (r ≈ 0.754), and the Conners Continuous Performance Test (r ≈ 0.773). Moderate correlations were also reported with ADHD-related inattention measures (r = 0.406–0.544). The instrument has demonstrated high diagnostic sensitivity, significantly predicting current and retrospective ADHD symptoms and supporting the ecological and construct validity of VR-based assessment.
- The Internet Addiction Test (IAT), developed by Young (1998), is a selfreport screening questionnaire that assesses different dimensions of problematic internet use, including time spent online, salience of internet use, difficulties in time management, perceived loss of control, and interference with daily functioning. The measure consists of 20 items rated on a five-point Likert scale, with total scores ranging from 20 to 100. Higher scores indicate greater severity of problematic internet use.
  IAT scores were interpreted according to commonly used severity ranges: 20–30 points indicate average internet use, 31–49 points mild problematic internet use, 50–79 points moderate problematic internet use, and 80–100 points severe problematic internet use. In this study, clinically relevant problematic internet use was defined as an IAT score ≥31, indicating at least mild problematic internet use. The IAT was therefore used as a dimensional self-report screening measure and not as a stand-alone diagnostic instrument. For this reason, the term “problematic internet use” is used throughout the manuscript, whereas “Internet Addiction Test” is retained only as the official name of the instrument.

The Italian validation study demonstrated strong psychometric properties, including high internal consistency (Cronbach’s *α* = 0.91) and adequate convergent and discriminant validity. Factor analytic findings supported a multidimensional structure reflecting compulsive use and functional impairment associated with problematic internet behavior (Fioravanti and Casale, 2015). Given the exploratory nature of the study and the limited sample size, psychometric parameters were not recalculated in the present dataset; therefore, previously established validation indices were reported.

### VR assessment procedure

VR assessment was conducted individually in a quiet clinical room at the Adult Neurodevelopmental Disorders Outpatient Clinic. Participants completed the Nesplora Aquarium task using the standard immersive VR equipment provided for the test administration. The same administration procedure was used at T0 and T1. Before starting the task, participants received standardized instructions and completed a brief familiarization phase to ensure that they understood the task requirements. The VR scenario and task sequence were repeated identically at follow-up. The assessment was administered by trained clinical personnel, and participants were monitored during the session for discomfort, fatigue, or cybersickness. No assessment was interrupted because of cybersickness or intolerance to the VR procedure. The headset used was Oculus Meta Quest 2. The total duration of the VR task was approximately 15 min.

### Statistical analysis

Given the small sample size and the distributional characteristics of the data, nonparametric methods were used. Paired comparisons between baseline (T0) and followup (T1) were performed using the Wilcoxon signed-rank test. Associations between IAT scores and VR-derived cognitive indices were examined using Spearman’s rank correlation coefficients separately at T0 and T1. Descriptive statistics are reported as means, standard deviations, medians, and interquartile ranges. Effect sizes for Wilcoxon signed-rank tests were calculated as r = Z/√N. To account for multiple testing, a false discovery rate procedure was applied when interpreting the results. Because the study was designed as an exploratory single-centre pilot investigation, no *a priori* power analysis was performed. Statistical analyses were conducted using SPSS® version 25, and Microsoft Excel® was used for descriptive calculations and graphical representation. The threshold for statistical significance was set at *p* < 0.05.

## Results

The study included 17 adults with clinically diagnosed ADHD and clinically relevant problematic internet use who completed both baseline and follow-up assessments. Descriptive analysis of medians and interquartile ranges indicates distinct performance profiles across the attention and executive functions, which corresponds to the neurocognitive profiles typical of ADHD.

At baseline (Table 1), working memory scores were quite low (Mdn = 37.0), and attention scores were at their highest (Mdn = 63.0). This pattern corresponds to the literature stating individuals with ADHD have greater deficits in executive functions relative to basic perceptual attentional skills.

**Table 1:** Descriptive statistics of neurocognitive variables (Nesplora Aquarium) and Internet Addiction Test (IAT) scores at baseline (T0).

Neurocognitive variables (Nesplora Aquarium) and Internet Addiction Test (IAT) scores	Mean	Standard deviation	Percentile 25	Percentile 50	Percentile 75
attention	62,1765	5,69,249	61,0000	63,0000	65,5,000
discrepancy	51,8,235	12,44,104	39,0000	55,0000	62,5,000
processing	35,8,235	7,54,350	30,5,000	33,0000	42,5,000
vigilance	57,3,529	11,32,994	51,0000	60,0000	64,5,000
inhibitory_control	59,0588	9,94,655	48,0000	60,0000	66,5,000
switching	55,7,647	13,60,850	44,5,000	59,0000	66,5,000
perseveration	64,1,176	6,71,642	59,0000	63,0000	71,0000
working_memory	37,0000	4,71,699	34,0000	37,0000	39,5,000
IAT	53,5,882	16,55,317	41,0000	47,0000	70,5,000

*N* = 17.

### Follow-up comparisons and correlations

**Figure 1:**
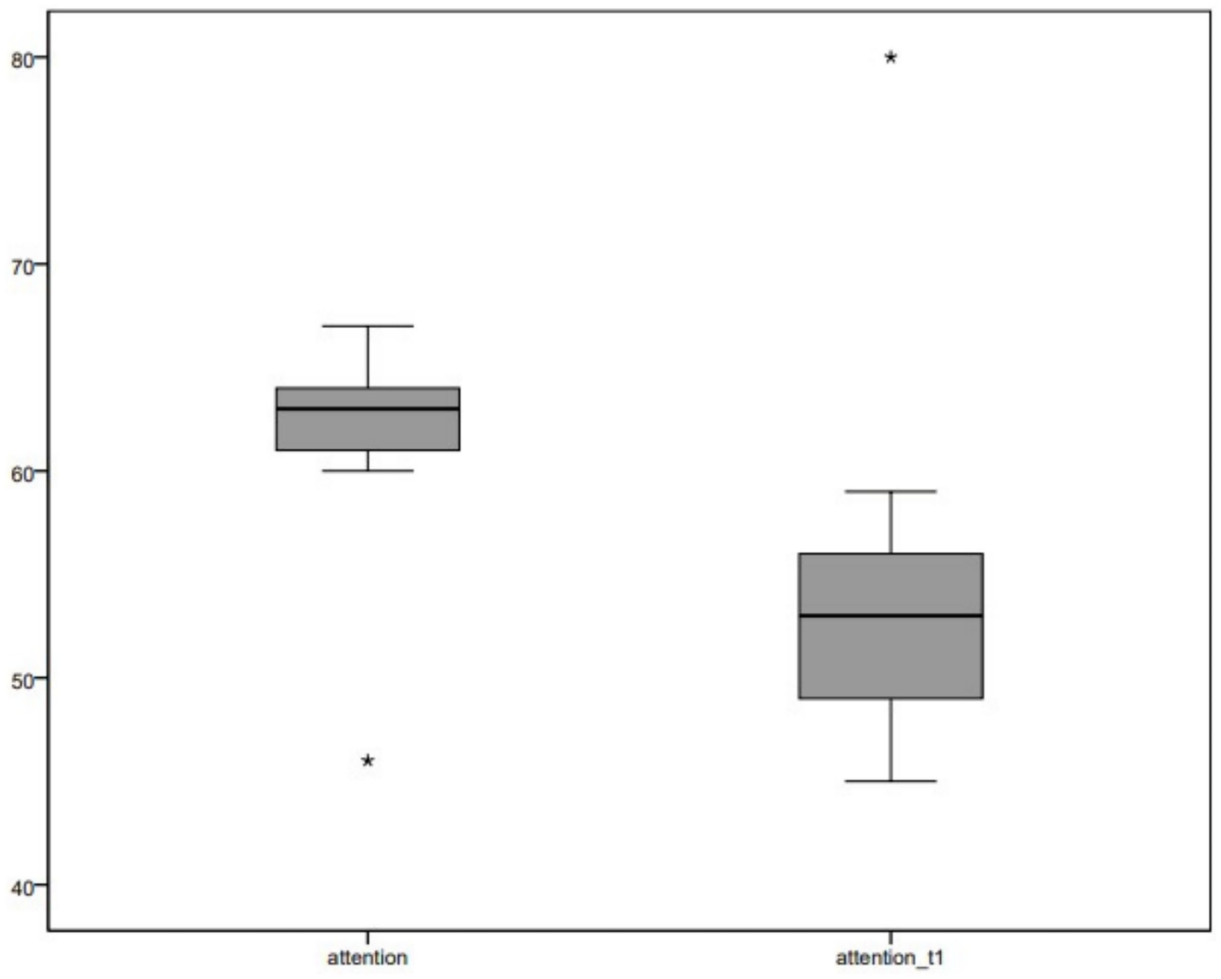
Boxplot comparisons of attention scores between baseline (T0) and follow-up (T1). The plot illustrates the distribution of attention scores before and after methylphenidate treatment. A statistically significant decline was observed at T1 (*p* = 0.004).

**Figure 2:**
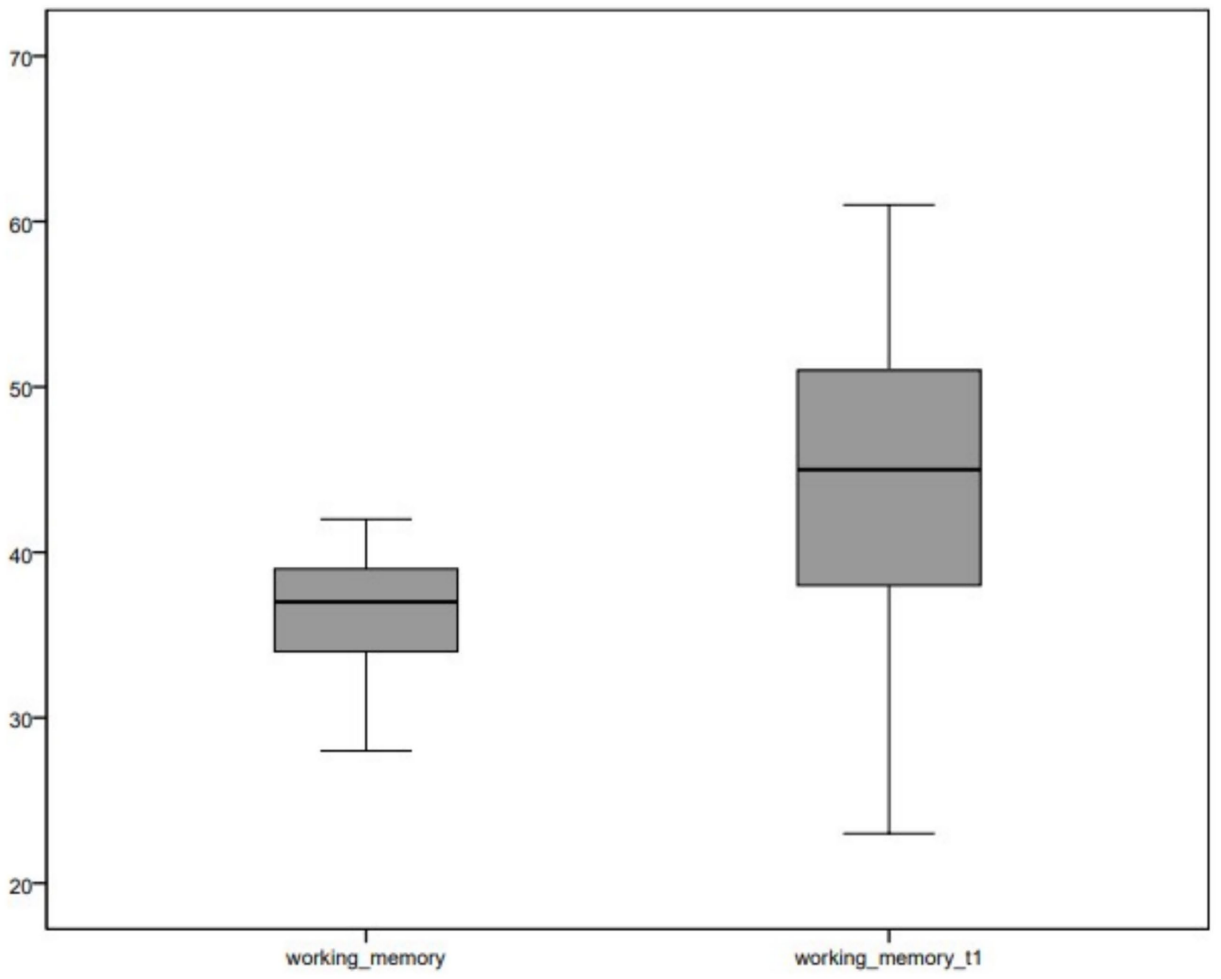
Boxplot comparisons of Working Memory scores between baseline (T0) and follow-up (T1). The plot illustrates the improvement in working memory performance after 4 weeks of treatment. The increase in scores was statistically significant (*p* = 0.008).

Statistically, the follow-up (Table 2) and baseline assessments showed changes in several cognitive domains after treatment, with the attention scores with the greatest decline (Z = −2.899, *p* = 0.004, large effect size), and working memory scores with the greatest improvement (Z = −2.638, *p* = 0.008, moderate to large effect size), which suggests differential changes across attentional and executive-function domains during the observation period. Before adjustment for multiple testing, additional changes were observed in vigilance (Z = −2.174, *p* = 0.030), perseveration (Z = −2.062, *p* = 0.039), and IAT scores (Z = −2.392, *p* = 0.017). After adjustment, the most robust findings were the decrease in attention scores and the increase in working memory scores, whereas the changes in IAT, vigilance, and perseveration should be interpreted as exploratory trends (Table 3; Figures 1, 2).

**Table 2:** Descriptive statistics of neurocognitive variables (Nesplora Aquarium) and Internet Addiction Test (IAT) scores at follow-up (T1) after 4 weeks of methylphenidate treatment.

Neurocognitive variables (Nesplora Aquarium) and Internet Addiction Test (IAT) scores	Mean	Standard deviation	Percentile 25	Percentile 50	Percentile 75
attention_t1	55,0588	9,38,397	48,5,000	53,0000	57,5,000
discrepancy_t1	49,5,882	7,14,194	43,5,000	48,0000	56,0000
processing_t1	36,7,059	9,36,593	31,0000	34,0000	45,0000
vigilance_t1	52,3,529	9,43,359	47,5,000	54,0000	56,0000
inhibitory_control_t1	56,9,412	12,08031	48,5,000	53,0000	65,5,000
switching_t1	52,4,118	7,35,747	46,5,000	52,0000	59,0000
perseveration_t1	56,1765	9,86,303	48,0000	60,0000	62,0000
working_memory_t1	44,0588	9,69,839	37,5,000	45,0000	52,0000
IAT_t1	44,9,412	16,48,283	33,5,000	38,0000	54,0000

**Table 3:** Results of the Wilcoxon signed-rank test comparing neurocognitive performance and problematic internet use severity between baseline (T0) and follow-up (T1).

Neurocognitive variables (Nesplora Aquarium) and Internet Addiction Test (IAT) scores	Z	r	Sig. Asint. 2 code (*p*-value)
attention_t1 - attention	−2.899ᵇ	0.70	**0.004**
discrepancy_t1 - discrepancy	−0.355ᵇ	0.09	0.722
processing_t1 - processing	−0.129ᵇ	0.03	0.897
vigilance_t1 - vigilance	−2.174ᵇ	0.53	**0.030**
inhibitory_control_t1 - inhibitory_control	−0.595ᵇ	0.14	0.552
switching_t1 - switching	−0.983ᵇ	0.24	0.326
perseveration_t1 - perseveration	−2.062ᵇ	0.50	**0.039**
working_memory_t1 - working_memory	−2.638ᶜ	0.64	**0.008**
IAT_t1 - IAT	−2.392ᵇ	0.58	**0.017**

^a^Wilcoxon test; ^b^Based on positive ranks; ^c^Based on negative ranks.

Value reported in bold are statistically significant at *p < 0.05.

Changes across cognitive domains were not uniform. The contrast between sustained attention and working memory suggests that short-term variations in adults with ADHD may differ across attentional and executive-function indices. Given the exploratory design, these findings should be interpreted as preliminary indicators of cognitive variability rather than as evidence of a specific treatment effect.

Correlational analyses conducted at the initial point failed to explain all relationships between cognitive functioning and the extent of problematic internet use. IAT scores were most significantly related in a positive way to working memory ability (*ρ* = 0.503, *p* = 0.040). However, this association should not be interpreted as evidence of a causal relationship. Given the exploratory nature of the study, it may reflect compensatory mechanisms or other unmeasured factors linking executive functioning and problematic internet use. Other correlations between IAT scores and other cognitive scores were minor or of no importance. This means the correlation with working memory is more of a unique case than of a general pattern (Tables 4, 5). At follow-up (T1), the repeat analyses showed no correlational relationships of any significance with the assessed variables. No significant correlations were observed at T1, suggesting that the association identified at baseline was not maintained at followup. Attention and working memory scores, along with other primary cognitive indices, signify sensitive cognitive markers for monitoring treatment changes in adults with both comorbid ADHD and problematic internet use. The changes in how cognitive and behavioral factors were correlated from the beginning to the follow up suggest the need for further analysis to explain the mechanisms behind such changes over time.

**Table 4:** Spearman’s rank correlation coefficients between Internet Addiction Test (IAT) scores and neurocognitive variables at baseline (T0).

Spearman’s Rho		IAT
attention	Correlation coefficient	−0.443
	Sig. (2-code)	0.075
	N	17
discrepancy	Correlation coefficient	0.136
	Sig. (2-code)	0.602
	N	17
processing	Correlation coefficient	0.162
	Sig. (2-code)	0.535
	N	17
vigilance	Correlation coefficient	−0.242
	Sig. (2-code)	0.349
	N	17
inhibitory_control	Correlation coefficient	0.062
	Sig. (2-code)	0.813
	N	17
switching	Correlation coefficient	0.332
	Sig. (2-code)	0.193
	N	17
perseveration	Correlation coefficient	0.033
	Sig. (2-code)	0.901
	N	17
working_memory	Correlation coefficient	0.503*
	Sig. (2-code)	**0.040**
	N	17
IAT	Correlation coefficient	1,000
	Sig. (2-code)	
	N	17

*p < 0.05 (2-code); **p < 0.01 (2-code).

Value reported in bold are statistically significant at *p < 0.05.

**Table 5:** Spearman’s rank correlation coefficients between Internet Addiction Test (IAT) scores and neurocognitive variables at follow-up (T1).

Spearman’s Rho		IAT
attention_t1	Correlation coefficient	−0.282
	Sig. (2-code)	0.273
	N	17
discrepancy_t1	Correlation coefficient	−0.207
	Sig. (2-code)	0.426
	N	17
processing_t1	Correlation coefficient	0.326
	Sig. (2-code)	0.202
	N	17
vigilance_t1	Correlation coefficient	−0.060
	Sig. (2-code)	0.818
	N	17
inhibitory_control_t1	Correlation coefficient	−0.273
	Sig. (2-code)	0.290
	N	17
switching_t1	Correlation coefficient	−0.128
	Sig. (2-code)	0.624
	N	17
perseveration_t1	Correlation coefficient	−0.166
	Sig. (2-code)	0.524
	N	17
working_memory_t1	Correlation coefficient	0.042
	Sig. (2-code)	0.872
	N	17
IAT_t1	Correlation coefficient	1,000
	Sig. (2-code)	
	N	17

No significant correlations were observed at T1 (*p* > 0.05). **p < 0.01 (2-code).

## Discussion

The present pilot study explored the sensitivity of virtual-reality–derived cognitive indices to short-term performance variations observed in adults with ADHD and clinically relevant problematic internet use during routine clinical treatment. The results showed divergent outcomes between attentional and executive domains, with a decrease in attentional performance and an increase in working memory. This pattern confirms the heterogeneity of neurocognitive deficits in ADHD, in which difficulties with vigilance and attentional stability can coexist with more marked executive deficits (Willcutt et al., 2005; Faraone et al., 2021).

The improvement in working memory observed at T1 occurred during the treatment period and is consistent with previous literature describing executive-function modulation under stimulant therapy, although causal interpretations cannot be inferred from the present study. Previous studies and meta-analytic evidence suggest that methylphenidate may improve selected executive-function domains, including working memory, through dopaminergic and noradrenergic modulation of frontostriatal and prefrontal networks (Fuermaier et al., 2017; Pievsky and McGrath, 2018; Quintero et al., 2022).

At the same time, the reduction in attention scores observed between T0 and T1 may reflect the known variability of attentional stability in ADHD, where sustained attention often shows greater intra-individual fluctuation compared with executive domains (Lufi et al., 2015). Although methylphenidate can improve attention levels, its effects appear to be more consistent on executive aspects than on attention stability, as demonstrated in the literature (Linssen et al., 2014; Rubia et al., 2014).

A relevant exploratory finding concerns the positive correlation between problematic internet-use severity, measured using the Internet Addiction Test (Young, 1998), and working memory at baseline. This finding suggests that relatively preserved executive functions may be associated with sustained engagement in cognitively demanding online activities; however, it should be considered exploratory and hypothesis generating.

This interpretation is consistent with the I-PACE (Interaction of Person–Affect– Cognition–Execution) model, according to which predisposing vulnerabilities interact with affective and cognitive responses and executive functions in sustaining addictive behaviors (Brand et al., 2016; Dong and Potenza, 2014).

In line with this picture, a recent multicenter study conducted in Germany (Müller et al., 2025) showed that online addictive behaviors are associated with generalized deficits in executive functions, including disadvantageous decision-making, reduced cognitive flexibility, and stimulus-specific inhibitory control deficits, independent of psychiatric comorbidities.

These findings highlight how executive dysfunctions represent a transdiagnostic marker of Internet use disorders, with particularly evident manifestations in areas such as compulsive shopping and problematic pornography use.

The positive association observed between working memory performance and problematic internet use could be clarified through further interpretation. More recent evidence indicates that some forms of problematic Internet use may be supported by relatively preserved or actively engaged cognitive resources, despite traditional models considering executive deficits to be a vulnerability factor for addictive behaviors. Activities associated with excessive Internet use, such as online gaming, prolonged browsing, or digital multitasking, require continuous information updating, careful monitoring, and goal maintenance; these processes depend primarily on working memory (Ioannidis et al., 2019).

Executive functions in the I-PACE model may help prevent harmful behaviors when cognitive resources are used to support reward-based online activities (Brand et al., 2016). From this perspective, people with good working memory may be more inclined to engage for long periods of time in complex digital environments, which facilitates the creation of compulsive usage patterns. Neurocognitive models describe behavioral addictions as the result of an ongoing imbalance between reward and cognitive control systems, rather than simply an impairment of executive functions in general. This interpretation is consistent with this theory (Dong and Potenza, 2014).

However, the disappearance of this association at T1, coinciding with drug treatment, indicates a possible rebalancing of the relationship between cognitive functions and dysfunctional use of technology. This trend is in line with the findings (Grassi et al., 2024), who reported a significant reduction in behavioral addiction symptoms, including problematic internet use, in adults with ADHD treated with methylphenidate. These findings suggest that the improvement induced by pharmacotherapy may contribute to reducing the link between executive resources and the maintenance of problematic online behaviors.

The effects observed in vigilance and perseveration were weaker and should be interpreted cautiously. These findings suggest that VR-derived indices may capture variability across different cognitive domains, although the small sample size and the absence of a control group limit any firm interpretation (Neguț et al., 2016; Satu et al., 2025).

These findings may suggest potential functional implications associated with improvements in executive functioning. Enhanced working memory and cognitive control may translate into greater efficiency in managing complex tasks, improved planning abilities, and more regulated behavioral responses, including a more controlled use of digital technologies. However, these interpretations should be considered cautiously, as functional outcomes were not directly assessed in the present study.

In daily functioning, these effects are expected to manifest as enhanced planning and organizational skills: the individual should become more capable of handling complex tasks without losing track or omitting intermediate steps. Although functional implications can be hypothesised, outcomes in daily life were not directly measured in the present study. Consequently, behavioral improvements resulting from cognitive changes in the real world should be interpreted with caution and require further investigation in future controlled research.

Overall, the results indicate that cognitive indices obtained from virtual reality may be related to short-term changes in attentional and executive performance observed in adults with ADHD receiving routine clinical treatment. Rather than demonstrating treatment efficacy, the present findings support the feasibility of virtual reality-based monitoring as a complementary assessment method that requires confirmation in larger controlled studies.

## Conclusion

The study highlighted the heterogeneity of cognitive profiles in adults with ADHD and problematic internet use, showing that alongside persistent vulnerabilities in attentional processes, there is a significant improvement in working memory after treatment with methylphenidate. This trend confirms that executive functions respond more favorably to pharmacotherapy than vigilance and sustained attention, which remain areas of fragility typical of the disorder.

A particularly interesting finding concerns the initial correlation between working memory and problematic online behaviors, which suggests a paradoxical role of executive resources in maintaining problematic internet use. However, this link seems to diminish with the introduction of drug therapy, indicating a possible rebalancing of the cognitive dynamics underlying these dysfunctional behaviors.

Furthermore, the use of virtual reality has proven to be an innovative and promising tool, capable of capturing cognitive changes with greater sensitivity and ecological validity, and potentially useful for guiding more personalized therapeutic pathways. In conclusion, these results emphasize that ADHD should be understood not as a simple attention disorder, but as a complex and multidimensional condition.

The integration of pharmacotherapy and advanced assessment tools, such as virtual reality, opens up important prospects for more targeted clinical management and the reduction of associated behavioral comorbidities.

## Limitations

This study has several limitations. First, the relatively small sample size, which is related to the pilot design, limits the generalizability of the results and limits the identification of possible gender-related differences. Future studies should confirm these findings in bigger and more heterogeneous samples.

Second, the repetition of the same virtual reality (VR) scenario at both time points may have introduced retest effects, that could influence attention consistency and response speed. The development of adaptive or alternative VR tasks could help minimize possible distortions in longitudinal studies. The exclusive use of self-assessment tools, such as the Internet Addiction Test (IAT), represents a further methodological limitation, as these tools can lead to distortions in responses and social desirability effects, which can reduce the objectivity of the data collected. Another limitation concerns the impossibility of examining psychometric properties within the present sample. Due to the limited sample size, reliability and factorial analyses were not recalculated. These procedures require substantially larger samples to produce stable estimates. The study’s instruments have all been shown to be reliable and valid in large groups of people in the past. Consequently, the interpretation of the findings relies on established psychometric evidence rather than sample-specific measurement parameters. A further limitation concerns the confounding influence of drug treatment. All participants were on methylphenidate therapy, and it was not possible to isolate drug-related cognitive improvements from other factors such as practice or placebo effects. The inclusion of untreated or non-pharmacological control groups would allow for a more accurate interpretation of the results. Furthermore, the relationship between problematic internet use and working memory, which was significant at baseline but attenuated at T1, should be interpreted with caution, as it may reflect a transient or compensatory mechanism rather than a stable association.

## Data Availability

The original contributions presented in the study are included in the article/supplementary material, further inquiries can be directed to the corresponding author.
